# His bundle pacing – a curative method: A Case Report

**DOI:** 10.1097/MD.0000000000021633

**Published:** 2020-08-07

**Authors:** Catalin Pestrea, Alexandra Gherghina, Florin Ortan, Gabriel Cismaru, Rosu Radu

**Affiliations:** a Department of Interventional Cardiology, Brasov County Emergency Clinical Hospital; b 5th Department of Internal Medicine, Cardiology-Rehabilitation, “Iuliu Hatieganu” University of Medicine and Pharmacy Cluj-Napoca, Romania.

**Keywords:** left bundle branch block, His pacing, electrode, threshold, sensing, AV block

## Abstract

**Introduction:**

Pacing of the His bundle and conduction system seems an attractive site for pacing. Lead placement in His-pacing might be technically challenging due to surrounding structures and particular anatomic location.

**Patient concerns:**

A 62-years old male patient was admitted for recurrent syncope. Electrocardiographic monitoring revealed periods of complete atrioventricular block with left branch block morphology and a QRS duration of 160 ms.

**Diagnosis:**

A diagnosis of intermittent complete atrioventricular block was made with a Class I indication of permanent dual-chamber cardiac pacing.

**Interventions:**

A lead delivery system with a C315 His catheter and a Select Secure 3830 69 cm pacing lead was placed at the septal area of the atrioventricular junction with good pacing and sensing thresholds. An important narowing of the QRS was observed.

**Outcomes:**

After the procedure, good pacing and sensing parameters were observed.

Echocardiography revealed disappearance of the previously recorded ventricular dyssynchronism.

Device follow-up at 1 month and 3 months showed stable pacing and sensing parameters.

**Conclusion:**

Pacing the distal His bundle normalized the QRS complex, therefore “curing” both the atrioventricular and the left bundle branch conduction abnormalities. As such, the technique can be used as an alternative to cardiac electrical resynchronization therapy with acceptable pacing and detection thresholds and better ventricular activation pattern.

## 1 Introduction

Cardiac pacing remains the only effective therapy for patients with symptomatic AV block or sinus node disease.^[1]^ To date there is a continuous debate on the optimal ventricular pacing site: apex, RVOT or septum.^[2]^ Biventricular pacing demonstrated improvement in patients with heart failure, reduced ejection fraction and left bundle branch block.^[3]^ However, in the last decades, stimulation of the His bundle emerged as a very attractive site for pacing due to ventricular activation through the intrinsic conduction system. Lead placement in His bundle pacing might be technically challenging due to surrounding structures and particular anatomic location. This is illustrated by our case report: the level of the block was intrahisian, therefore the stimulation of the distal His bundle normalized the QRS complex and synchronized the left ventricular activation.

## 2 Case report

A 62-years old male patient was admitted to the Cardiology Department of the Brasov County Emergency Clinical Hospital for recurrent syncope. The patient‘s medical history included arterial hypertension, mild valvular regurgitations and nonsignificant coronary artery stenosis. Electrocardiographic monitoring revealed periods of completely intermittent atrioventricular block with left branch block morphology and a QRS duration of 160 ms. Laboratory values showed no reversible causes of AV block. Echocardiography revealed intra and interventricular dyssynchronism, with a preserved ejection fraction.

After thorough cardiological examination, the patient was considered to have Class I indication for permanent dual-chamber cardiac pacing and was proposed for permanent pacing of the His bundle.

**Figure 1:**
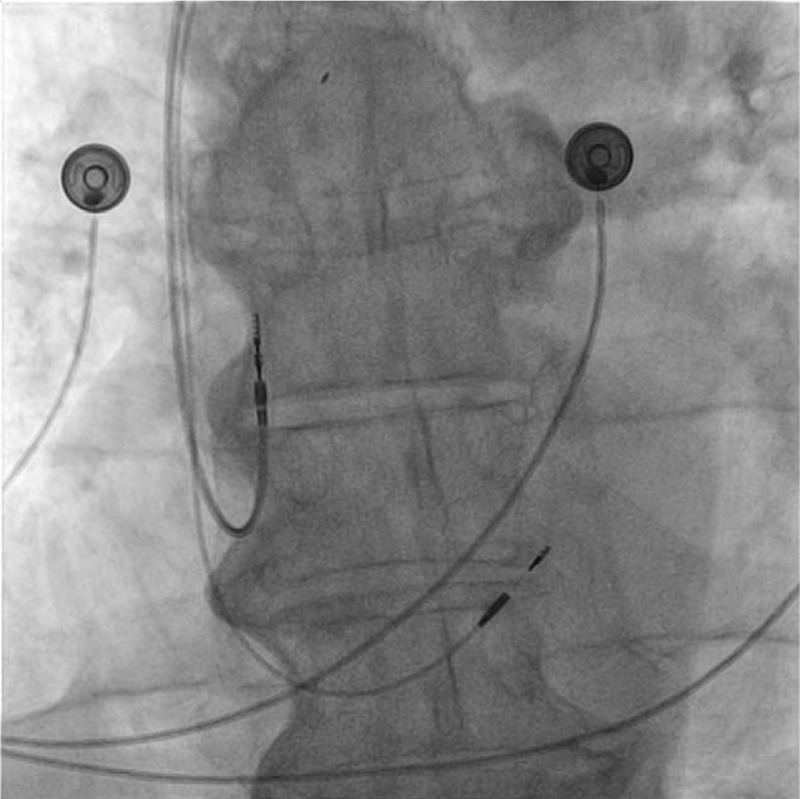
Fluoroscopic antero-posterior view of the atrial and ventricular leads implanted inside the right heart chambers: both leads are active fixation leads. The superior lead is fixated at the level of right atrial appendage and the inferior lead at the level of His bundle.

**Figure 2:**
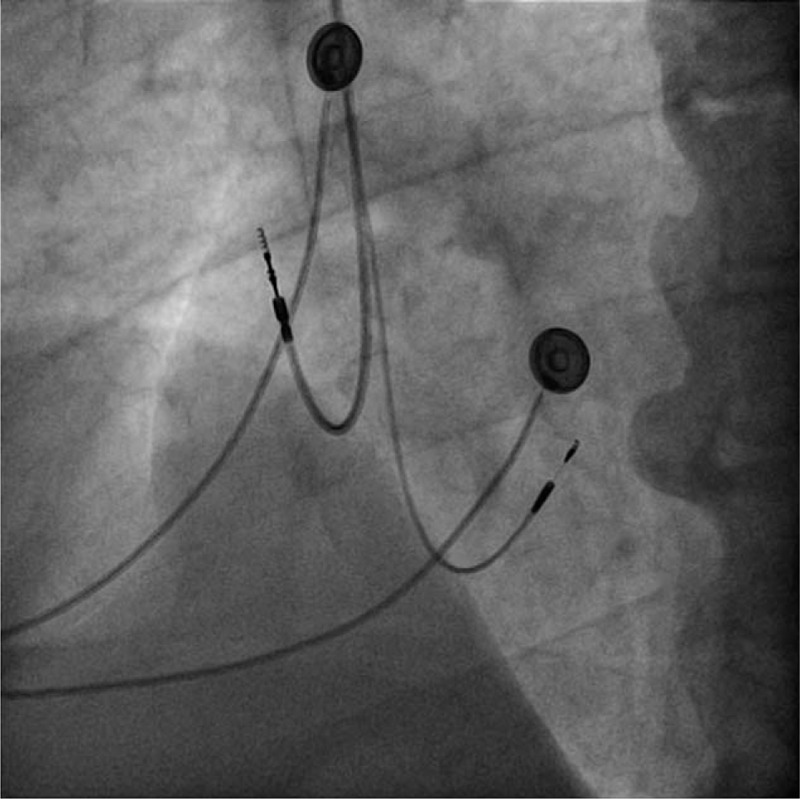
Fluoroscopic left anterior oblique view of the atrial and ventricular leads implanted inside the right heart chambers: both leads are active fixation leads. The superior lead is the atrial lead and is oriented towards the lateral wall of the right atrium, in contrast with the inferior lead which is the His-bundle pacing lead oriented towards the interventricular septum.

**Figure 3:**
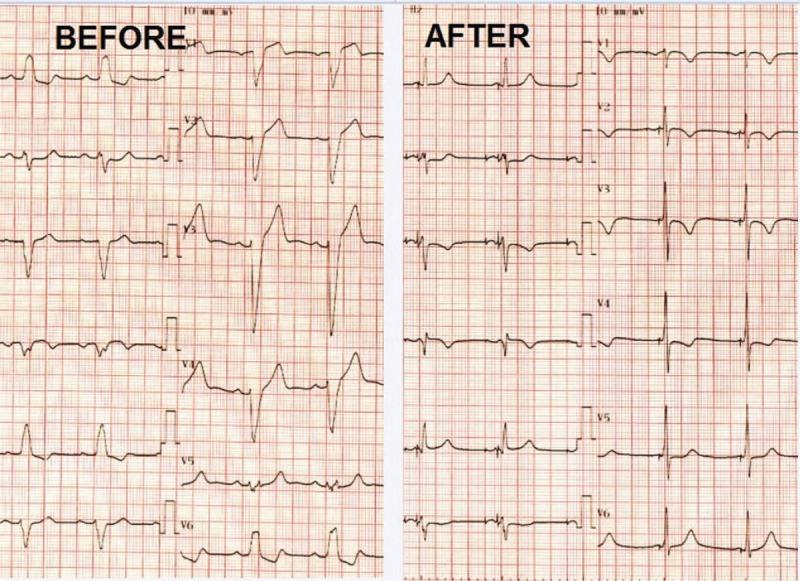
Twelve-lead ECG before and after His-pacing: Before pacing ECG shows a left bundle branch block morphology with a large QRS of 120 ms. After pacing ECG shows atrial and ventricular pacing with a narrow QRS of 90 ms. ECG = electrocardiogram.

A lead delivery system consisting of a C315 His catheter (Medtronic Inc., Minneapolis, MN, USA) and a Select Secure 3830 69 cm pacing lead (Medtronic Inc., Minneapolis, MN) was placed at the septal area of the atrioventricular junction (Figs. 1 and 2). In that region endocardial mapping was performed in a unipolar setting using the lead tip until a distal His bundle signal was obtained. At this level, pacing at variable amplitudes showed nonselective His bundle capture with important narrowing of the paced QRS complex (Fig. 3). The lead was fixed at this level with a final right ventricle pacing threshold of 0.25 V / 1 ms and a non-selective His capture threshold of 1,5 V/L ms with complete correction of the left bundle branch block and a paced QRS duration of 90 ms. Detection was measured at 5.2 mV.

The post-procedural echocardiographic reassessment revealed the disappearance of the previously recorded dyssynchrony parameters.

Device follow-up at 1 month and 3 months showed stable pacing and sensing parameters of the His bundle pacing lead: 1.5 V /Lms, respectively 5 mV, the patient being completely asymptomatic.

## 3 Discussion and Conclusion

In patients with LBBB and mild myocardial impairment (FE > 35%), there is data showing that they have a higher mortality rate than those without conduction disorders and a higher risk of long-term progression to heart failure.^[4]^ These patients were not included in the large trials of cardiac resynchronization therapy and therefore currently there are no device intervention recommendations in these cases.

However, when there is an indication for conventional cardiac pacing, such as sinus node disease or symptomatic atrioventricular block, and the patient associates left bundle branch block, it is intuitive that electrical resynchronization of the heart, both atrioventricular and interventricular, would have long-term benefits.

Cardiac resynchronization therapy (CRT) by biventricular stimulation is currently the gold standard of electrical and mechanical resynchronization. Patients with LBBB and low ejection fraction benefit from CRT with an acute hemodynamic improvement that is translated in reduced morbidity and mortality.^[5,6]^ However, the classical therapy of cardiac resynchronization, which involves endocardial stimulation of the right ventricle and epicardial stimulation of the left ventricle, which might be effective in some patients, is far from being a physiological therapy, and this is observed from the morphology of the paced QRS complex (negative in lead I, positive in V1, most often with a duration longer than 120 ms). In fact, biventricular stimulation induces intraventricular and interventricular dissynchronism if applied to patients that have a narrow QRS which is further translated into worse clinical outcome^[7,8]^ Furthermore, there is electrophysiological evidence that the site of block in LBBB might be at the level of the His bundle or in the proximal portion of the left bundle branch.^[9]^ Therefore, it would be possible to capture the conduction system beyond the level of block, with restoration of the normal conduction of electrical impulses.

In our case report, the level of block was intrahisian and the stimulation of the distal His bundle normalized the QRS complex. Thus, the technique can be used as an alternative to cardiac resynchronization therapy with acceptable pacing and detection thresholds and better results in ventricular activation pattern.^[10]^

The recently reported His-SYNC pilot trial showed promising results in patients with LBBB and heart failure with His bundle pacing being non-inferior to biventricular pacing in terms of improvement in left ventricular ejection fraction, cardiovascular hospitalization or death.^[11]^ There are some potential drawbacks to this procedure. Due to the limited availability of specialized delivery systems, reaching the His bundle position could be difficult, especially in enlarged cardiac chambers and displaced His bundle. Furthermore the lead tip position at the AV junction could lead to either atrial oversensing or ventricular undersensing. There are also reports of increasing pacing thresholds in time and a possible progression to a more distal conduction disease which would make His bundle pacing redundant, but overall these issues seem to occur rarely.^[12]^ Also, macrodislodgements of the lead have been rarely reported. Taken the above under consideration, patient selection is important in achieving procedural success. Older patients with enlarged cardiac chambers, ischemic heart disease (which usually means diffuse distal conduction disease) and severe comorbidities and with electrophysiological evidence of infrahisian disease are less likely to benefit from this procedure.^[13]^ Nevertheless, if properly achieved, His bundle pacing can normalize the cardiac electrical conduction pattern with less lead burden and lead related complications. This was our case, in which fortunately the patient had an intrahisian block, which was “cured ” by direct pacing of the conduction system just beyond the line of block. Conclusion: Pacing the distal His bundle normalized the QRS complex, therefore “curing” both the atrioventricular and the left bundle branch conduction abnormalities. As such, the technique can be used as an alternative to cardiac electrical resynchronization therapy, with acceptable pacing and detection thresholds and better ventricular activation pattern.

**Consent:** Informed written consent was obtained from the patient for the publication of this case report and any accompanying medical images.

## Author contributions

**Resources:** Catalin Pestrea, Alexandra Gherghina, Florin Ortan.

**Supervision:** Catalin Pestrea, Cismaru Gabriel, Radu Rosu

**Visualization:** Catalin Pestrea, Alexandra Gherghina, Florin Ortan, Cismaru Gabriel, Radu Rosu

**Writing – original draft:** Catalin Pestrea, Cismaru Gabriel

**Writing – review & editing:** Catalin Pestrea, Cismaru Gabriel, Alexandra Gherghina, Florin Ortan, Radu Rosu.
